# Machine learning analysis of exome trios to contrast the genomic architecture of autism and schizophrenia

**DOI:** 10.1186/s12888-020-02503-5

**Published:** 2020-02-28

**Authors:** Sameer Sardaar, Bill Qi, Alexandre Dionne-Laporte, Guy. A. Rouleau, Reihaneh Rabbany, Yannis J. Trakadis

**Affiliations:** 1 Department of Human Genetics, McGill University, Montreal, QC Canada; 2 Department of Neurology and Neurosurgery, Montreal Neurological Institute, McGill University, Montreal, QC Canada; 3 School of Computer Science, McGill University, Montreal, QC Canada; 4 Montreal Institute for Learning Algorithms, Université de Montréal, Montreal, QC Canada; 5 Department of Medical Genetics, McGill University Health Center Room A04.3140, Montreal Children’s Hospital,1001 Boul. Décarie, H4A 3J1 Montreal, Quebec Canada

**Keywords:** Genomic, Machine learning, Unsupervised clustering, Autism spectrum disorder, Schizophrenia

## Abstract

**Background:**

Machine learning (ML) algorithms and methods offer great tools to analyze large complex genomic datasets. Our goal was to compare the genomic architecture of schizophrenia (SCZ) and autism spectrum disorder (ASD) using ML.

**Methods:**

In this paper, we used regularized gradient boosted machines to analyze whole-exome sequencing (WES) data from individuals SCZ and ASD in order to identify important distinguishing genetic features. We further demonstrated a method of gene clustering to highlight which subsets of genes identified by the ML algorithm are mutated concurrently in affected individuals and are central to each disease (i.e., ASD vs. SCZ “hub” genes).

**Results:**

In summary, after correcting for population structure, we found that SCZ and ASD cases could be successfully separated based on genetic information, with 86–88% accuracy on the testing dataset. Through bioinformatic analysis, we explored if combinations of genes concurrently mutated in patients with the same condition (“hub” genes) belong to specific pathways. Several themes were found to be associated with ASD, including calcium ion transmembrane transport, immune system/inflammation, synapse organization, and retinoid metabolic process. Moreover, ion transmembrane transport, neurotransmitter transport, and microtubule/cytoskeleton processes were highlighted for SCZ.

**Conclusions:**

Our manuscript introduces a novel comparative approach for studying the genetic architecture of genetically related diseases with complex inheritance and highlights genetic similarities and differences between ASD and SCZ.

## Background

Autism spectrum disorder (ASD) is a neurodevelopmental disorder characterized by significant impairments in social communication and interaction, as well as by abnormal repetitive behaviors, interests, or activities (Diagnostic and Statistical Manual of Mental Disorders (DSM)-5, 2013). The heritability of ASD has been estimated to be around 70–90%, suggesting that genetic factors contribute largely to the ASD phenotype [1]. Genome-wide sequencing analyses have revealed that a large number (100 to 1000) of susceptibility genes are associated with ASD [2–4]. Recent studies showed that de novo mutations (DNM) have a significant role in ASD [3, 5–7], and estimated that around 30% of simplex ASD cases result from DNMs [3].

Schizophrenia (SCZ) is a neuropsychiatric disorder characterized by distorted perception, emotion, and cognition. It can also be characterized by negative symptoms, such as anhedonia, blunting of affect, or poverty of speech and thought (DSM-5, 2013). Similar to ASD, SCZ has high heritability, estimated to be around 80–85%, yet, much of it is not fully understood [8]. Recent studies have highlighted a role for common single nucleotide polymorphisms (SNPs) in SCZ [9–12]. Moreover, like ASD, SCZ cases are enriched in de novo single nucleotide variants (SNVs) [13, 14].

In summary, both SCZ and ASD clearly have a strong genetic component in their etiopathology; however, linkage analysis and genome-wide associations have had limited success and replicability in identifying significant genes in these complex disorders [15–19]. The lack of success is thought to be due to ASD and SCZ having polygenic and multifactorial inheritance where, unlike Mendelian disorders, each susceptibility gene increases one’s predisposition to the disease in combination with other genes. The involvement of many genes (in different combinations for each patient) and environmental factors makes it difficult to identify the specific genetic risk factors predisposing a given patient to ASD or SCZ.

Machine learning (ML) or statistical learning (SL) algorithms aim to learn and understand complex high-dimensional data. These learning algorithms can be divided into two broad categories: supervised learning and unsupervised learning [20–22]. Our group recently applied supervised ML to rare, predicted functional variants from whole-exome sequencing (WES) data of a SCZ case-control dataset (*n* = 5090). 70% of the data was used to train the ML algorithm and 30% (*n* = 1526) to evaluate its performance, showing encouraging results (86% accuracy, *AUC*: 0.95) [23]. Studies based on supervised learning, like the one just mentioned, are focused on learning from input-to-output labeled data where a model is trained to learn the best function or map from input variables of data instances to their labels. In contrast, unsupervised learning algorithms seek to discover useful underlying patterns in a dataset without relying on labels. For instance, a recent publication using unsupervised learning illustrated how WES data could be used to identify patient subtypes of patients with major depressive disorder (MDD) [24].

Several studies have shown the effectiveness of supervised learning methods in distinguishing between overlapping medical conditions. For example, they have been used to distinguish between age-related cognitive decline and dementias based on neurocognitive tests [25]. Further, they have also been successfully used to distinguish and study different cancer types based on gene expressions [26, 27] and DNA methylation patterns [28].

Overlapping genetic factors conferring risk to both SCZ and ASD have been identified suggesting shared biological pathways [29]. Our hypothesis is that ML methods can help us advance our understanding of the genomic architecture of ASD and SCZ by contrasting exome data from patients with these two conditions. Analyzing data of individuals affected with two different conditions with high heritability, complex inheritance, and evidence for overlapping genetic features using supervised learning may have some advantages. For example, in our above-mentioned SCZ case-control study, some unaffected individuals may also be genetically at high risk for SCZ but not have been exposed to adequate environmental risk factors, complicating the analysis. When comparing individuals with ASD and SCZ, given they are all *affected*, this is not an issue anymore. The first objective of our study is to explore whether SCZ and ASD patients can be distinguished based solely on supervised learning analysis of the genetic information from their WES data. Our second objective is to analyze the genetic features prioritized by the supervised learning algorithm, using unsupervised clustering, to identify central hub genes in the genetic architecture for SCZ and ASD.

## Materials

### Whole-exome data sources and annotation

#### Schizophrenia WES data (dbGaP trios)

This dataset is available in the dbGaP (study phs000687.v1.p1). The samples in this dataset were collected from the University Hospital Alexander in Sofia, Bulgaria. Individuals with intellectual disability were excluded. Unrelated families with parents who did not have schizophrenia participated in the original study. Overall, 598 trios were included in our analysis.

#### Autism WES data (NDAR trios)

The data for 2392 families with ASD were obtained from NDAR (doi: 10.15154/1169318; doi: 10.15154/1169195). The original sequencing data is of families in the Simons Simplex Collection [30]. The proband had to: 1) be at least 36 months of age, 2) have a nonverbal IQ or nonverbal mental age of 24 months for children aged between 36 and 83 months, or 30 months for children aged 84 months and above, 3) not have a known genetic disorder, and 4) not have extensive birth complications such as prematurity and cerebral palsy. Moreover, one of the requirements for participation in the study was that both biological parents had to be willing to participate and that they should not have ASD.

### Summary of variant filtering criteria

Filtering was run through the rows of variants in each dataset so that only variants that met the following criteria were included in our analysis.

We selected for *coding variant types* annotated as “frameshift_deletion”, “frameshift_insertion”, “frameshift_substitution”, “nonsynonymous_SNV”, “stopgain”, or “stoploss,” and *variant functional types* annotated as “exonic”, “exonic_splicing”, or “intronic_splicing.” Furthermore, the selected variants had a minor allele frequency (MAF) equal to or less than 0.01. Lastly, on a per-individual basis, for variants to be called they needed to have a minimum number of 4 variant reads, a minimum depth of sequencing of 10 reads, and a minimum genotype quality of 90.

The selected variants were then arranged in a tabular format, where each row corresponded to a different individual. The clinical status (ASD vs SCZ) for each individual was denoted in the first column, while the variants meeting our criteria for each individual were denoted as separate columns, with values of 0, 1 or 2 in the corresponding cells indicating wildtype, heterozygous, and homozygous status for each selected variant for the respective individual.

## Methods

### Population stratification adjustment

A major confounder in the analysis of cross-origin datasets like the ones we are using is the population stratification due to differences in ancestry. Due to population structure, the ML algorithm could focus on SNVs unrelated to the disease, which are specific to the population from which the affected individuals originate. Our focusing on *rare* variants minimizes the impact of differences in population structure between the two datasets. However, to formally address this possibility, we implemented a well-established population stratification correction method for genome-wide data [Eigenstrat] [31]. Eigenstrat is based on the adjustment of the original SNVs data based on any population structure discovered using principal components analysis. We applied this approach to adjust for population differences between the ASD (NDAR trios) and the SCZ (dbGaP trios) datasets. To remove the population structure from our dataset, we used the top 4 axes of variation from Eigenstrat that were significant. This is expected to account for most of the population structure. Then we regressed each SNV or feature of our dataset on the four axes of variation and took its residuals to be the adjusted SNV values of our adjusted dataset that corrects for population structure. We adjusted the phenotype values in a similar fashion. Lastly, each adjusted genotype and phenotype value was rounded to the nearest whole number to estimate the nearest adjusted genotype and phenotype. As a result, the original binary class of ASD and SCZ was converted to integer values, which we then capped to a range of − 4 to + 4 as only one adjusted instance fell outside this range.

This dataset has the adjusted genotype values of each SNV arranged in columns for each row of patient sample and will be referred to as the *SNV-based* data*.* We also converted the adjusted SNVs datasets into “gene-level SNV counts” by summing together all adjusted SNVs values located in the same gene of any given patient. This dataset has the sums for each gene arranged in columns for each row of patient sample and is referred to as the *gene-based* data.

### Algorithm selection

Many powerful ML algorithms render themselves uninterpretable, making it difficult to understand their decision-making process. Trying to balance interpretability with model performance, we used a more interpretable state of the art ML algorithm: regularized gradient boosted machine (GBM) (XGBoost implementation) [32], which we also demonstrated as an effective algorithm in our previous study [23].

Regularized GBM is state of the art and has been proved successful in a wide range of tasks. Its highly regularized methodology of feature selection and ranking of features based on their relative importance in making accurate predictions made it a great candidate for our study. Of note, a regularized algorithm penalizes itself for complexity, and thus uses only features that are relevant and brings more intelligence to its architecture than complexity. In our study, this means using only genes that have high predictive power in combination with other genes, and discard the less informative ones, thereby reducing the number of candidate genes.

### Training the boosted regression trees models

Since the population structure adjusted datasets following the Eigenstrat methodology have continuous phenotype labels, we trained the boosted regression trees variant of GBM to predict the continuous label values of ASD and SCZ cases based on the *SNV-based* data and the *gene-based* data. Since the focus of this analysis is to classify patients as either ASD or SCZ, we framed the regression problem as a classification to allow for measurement of the prediction accuracy. We performed the following mapping of the continuous predicted value to the binary classes. Since the adjusted phenotype values for ASD cases all had values of 1 or greater, and the adjusted phenotype values for SCZ all had values of − 1 or lower, any prediction above 0 was mapped to a prediction of ASD class and any prediction below 0 was mapped to a prediction of SCZ class.

Given our ASD and SCZ datasets contain an unbalanced number of individuals, we decided to use a balanced approach by selecting an equal number of ASD and SCZ cases. This change ensured that accuracy would be a good measure of model performance. To this end, the first 598 samples were selected from the ASD cases to balance the two datasets. We trained and fine-tuned the boosted regression trees using 70% of the data (419 ASD vs. 419 SCZ samples) as a training and validation dataset. We then inspected the best performing model on the remaining, previously unseen, 30% of the data (*test dataset*; 179 ASD vs. 179 SCZ samples). The SNVs used by the *SNV-based* model were extracted and mapped to their corresponding genes to get the list of the most important genes. The most important genes used by the best *gene-based* model were also extracted.

In addition to the 70:30 split for evaluation, we also assessed the performance of a five-fold cross-validation using the whole dataset (598 ASD vs. 598 SCZ samples) to provide a comprehensive validation of the algorithm.

### Identification of genes central to ASD and SCZ

To find which genes are important to SCZ or ASD, and which of these genes appear to be mutated concurrently in affected individuals, a novel unsupervised clustering analysis was performed. The genes identified by the 1) *SNV-based* algorithm and 2) *gene-based* algorithm were compared, and the ones identified by both algorithms (*the overlapping ML list of genes*) were used for the subsequent analyses.

To identify the (networks of) genes important to SCZ, hierarchical clustering was performed for *the overlapping ML list of genes*, using only the SCZ cases and the *gene-based* dataset. The Jaccard coefficient was used as the similarity measure for clustering the genes. The Jaccard coefficient between any two genes was calculated as the number of shared SCZ cases having an SNV count value greater than 0 in *both* genes divided by the number of SCZ cases having an SNV count value greater than 0 in *either* gene. Gene distances were derived as one minus the Jaccard coefficient. Hierarchical clustering is performed based on the distances using Ward’s linkage method [33], which recursively joins elements and/or clusters to form new clusters while minimizing the increase in the variance of the new cluster. Lastly, a dendrogram showing clusters of similar genes based on the distance metric and linkage method was created. To determine the most important cluster of genes for SCZ, we applied the following approach.

For each *gene cluster* identified, the *number of genes* was counted (a). Similarly, the number of unique SCZ cases carrying a genetic change in at least one of these genes was determined (b). This number (b) represents the number of SCZ cases having a genetic variation in at least one of the genes in a given cluster. Then, by dividing (b) over (a) a ratio, specific for each cluster, was calculated. The cluster with the highest ratio was selected as the one containing genes central to SCZ, as it involved genes highly mutated, in different combinations, among the highest proportion of SCZ patients in our dataset.

The same analysis above was then repeated separately based on ASD cases to obtain the genes central to ASD.

### Analysis software

The “xgboost” (version 0.90.0.1) package [34] for R was used as the implementation of the XGBoost algorithm. The “scipy” (version 1.0.1) package [35] for Python was used for the hierarchical clustering analyses.

## Results

For our boosted regression trees models, we obtained an accuracy of 86% for the *SNV-based* model and 88% for the *gene-based* model. Detailed metrics of model performance are listed in Table 1. A five-fold cross validation was also performed to provide additional validation. Overall, the average validation accuracy over all five folds was 88% for both the *SNV-based* model and *gene-based* model (Table 2). The performance over cross-validation is consistent with the results from the single-fold training-validating with independent testing approach mentioned above.

**Table 1:** Performance of different approaches (algorithms) on test data

Method	Accuracy	Precision	Recall	NIR	*P*-value (Acc > NIR)	95% CI
SNV-based	0.86	0.73	0.98	0.63	< 4.97e-22	(0.82,0.89)
Gene-based	0.88	0.80	0.96	0.58	< 3.09e-36	(0.85,0.92)

The performance between the two algorithms trained to distinguish ASD cases from SCZ cases is measured on a previously unseen test dataset. The accuracy is a measure of the number of correctly predicted samples divided by the total number of samples

*Acc* Accuracy, *NIR* No information rate, *CI* Confidence interval

**Table 2:** Performance of SNV and Gene-based approaches using five-fold cross validation

Method	Accuracy	Precision	Recall	NIR	*P*-value (Acc > NIR)	95% CI
SNV-based	0.88	0.78	0.97	0.59	< 2.2e-16	(0.86,0.90)
Gene-based	0.88	0.81	0.95	0.57	< 2.2e-16	(0.86,0.90)

The performance between the two algorithms trained to distinguish ASD cases from SCZ cases is measured using five-fold cross validation. All performance metrics are the average of the five cross validation folds

*Acc* Accuracy, *NIR* No information rate, *CI* Confidence interval

The ten most important genes from the *gene-based* model and the *SNV-based* approach (including the actual SNV in parenthesis) are shown in Table 3. The *SNV-based* model utilized 322 SNVs, located in 313 unique genes. The *gene-based* model utilized 1845 genes. Combining the top 10 genes from both approaches yielded a total list of 16 genes (Supplemental Table 1), with an overlap of 4 genes including the top 2: SARM1 and QRICH2, and PCLO and PRPF31. Overall, out of all the genes used by both models, 151 genes were *overlapping* (Supplemental Table 2).

**Table 3:** Top 10 important genes from *SNV-based* and *gene-based* models

SNV-based approach (SNV rsID)	Gene-based approach
SARM1 (rs71373646)	SARM1
QRICH2 (rs6501878)	QRICH2
AKAP1 (rs34535433)	PRPF31
PCLO (rs77721383)	SEC24D
TSPO2 (rs147405274)	SCN4A
ABCC3 (rs11568605)	CACNA1S
KIF13A (rs41267712)	CDSN
FAN1 (rs150393409)	HERC2
CCDC155 (rs201671744)	MUC16
PRPF31 (rs199870856)	PCLO

Boosted regression trees models were trained to separate SCZ and ASD probands based on the population-structure-adjusted *SNV-based* and *gene-based* datasets. The 10 most important genes from the *gene-based* model, but also from the *SNV-based* approach (including the actual SNV in parenthesis), are shown in this table. The table is ordered from most to least importance

**Fig. 1:**
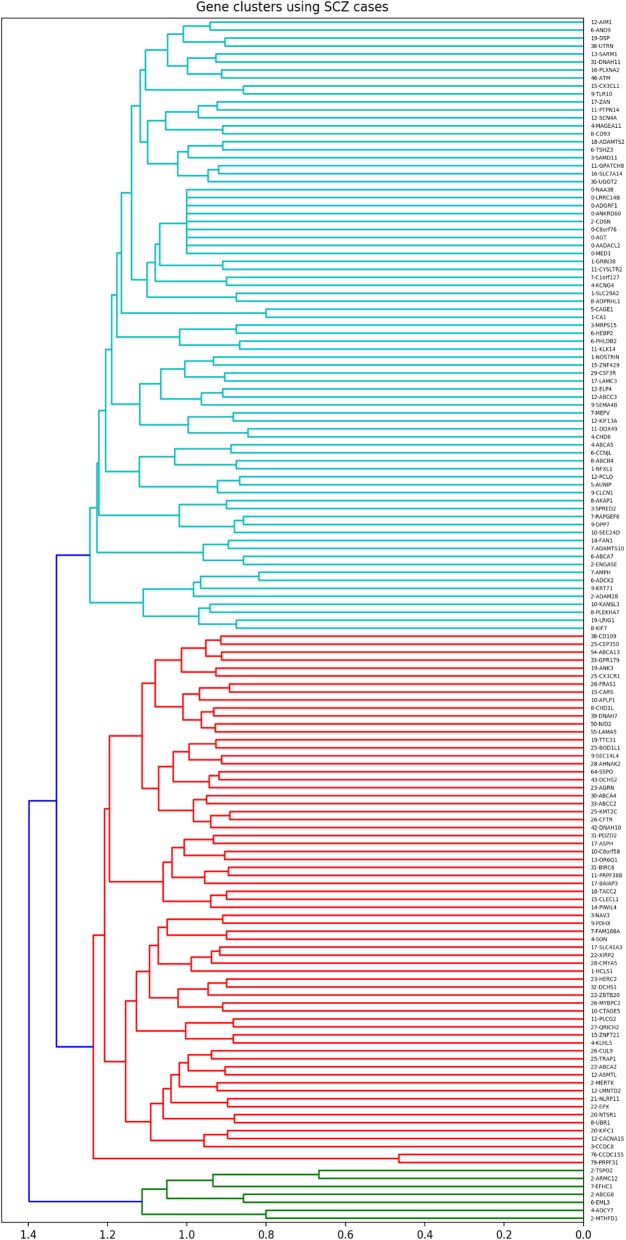
Hierarchical clustering of overlapping genes using SCZ cases

**Fig. 2:**
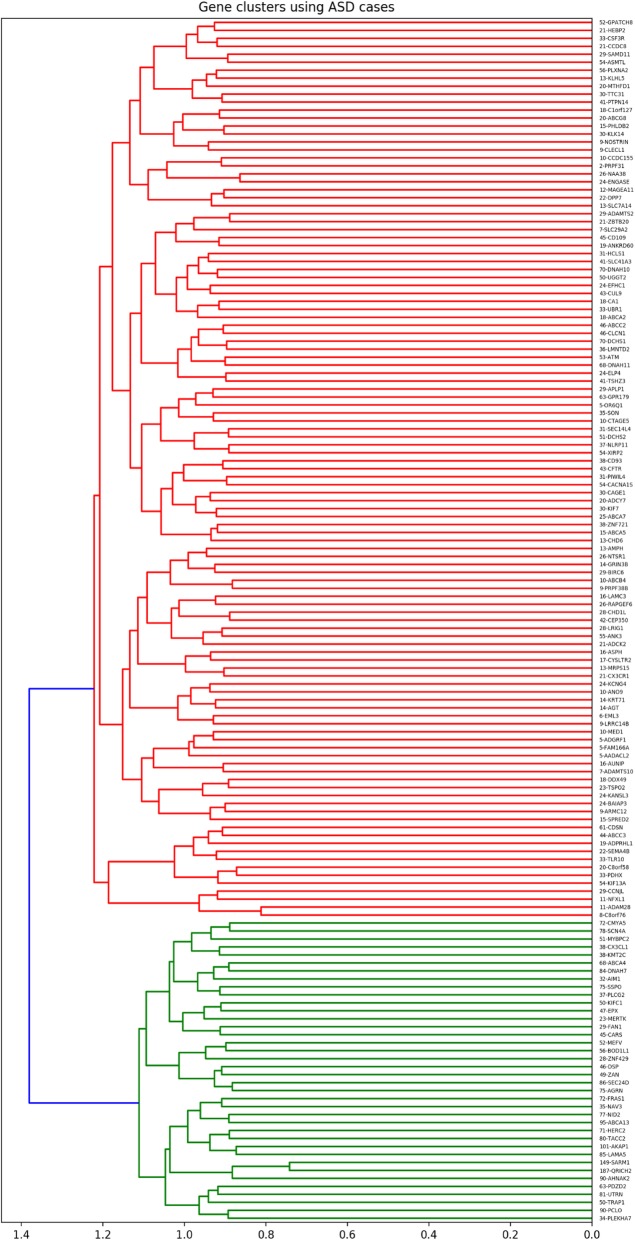
Hierarchical clustering of overlapping genes using ASD cases

Clustering of these 151 *overlapping* genes based on SCZ cases revealed three clusters of genes. Out of the three clusters, cluster 2 showed the highest ratio (7.55) of SCZ cases per cluster gene. Overall, 84.62% (506/598) of SCZ cases in our dataset had a genetic change in at least one of the genes in SCZ cluster 2, which is composed of 67 genes (Fig. 1, Supplemental Table 3). Similarly, clustering of the 151 *overlapping* genes, from Supplemental Table 2, based on ASD cases, revealed two clusters of genes. The highest ratio of cases per gene was 15.5 from ASD cluster 2. Overall, 98.49% (589/598) of ASD cases in our dataset had a genetic change in at least one of the genes in ASD cluster 2, which is composed of 38, out of the 151 *overlapping* genes being targeted (Fig. 2, Supplemental Table 4).

## Discussion

We have explored the genetic architecture of SCZ and ASD families through boosted regression trees (XGBoost) and clustering. Our focusing on *rare* variants minimizes the impact of differences in population structure between the two datasets. However, before performing any analyses, we also used the well-known Eigenstrat method to correct for any differences between datasets due to population structure. Overall, through boosted regression trees, we were able to find SNVs (and genes) which can distinguish between SCZ and ASD case status with accuracies of 88% for cross-validation and 85–90% on testing data (specifically, 86% for *SNV-based* method and 88% for *gene-based* method). To further study the important genes identified from the boosted regression trees, we hierarchically clustered the 151 genes identified from both algorithms (Supplemental Table 2) using only SCZ cases (and repeated the process for ASD). Our hypothesis was that some of the genes identified as part of the boosted regression trees approach might be important, central “hubs” for SCZ (and/or ASD). Through clustering of the 151 *overlapping* genes, based on the shared proportion of cases between genes, we were able to find groups of genes that were often mutated together in SCZ cases (and ASD cases, respectively).

Overall, we have demonstrated a novel approach for studying (comparing) the genetic architecture and pathophysiology of two diseases. Instead of using all SNVs from WES data, we first utilized a regularized machine learning approach optimized for large feature sets to identify the most important genes for separating the two groups (ASD and SCZ in this case). This step can potentially reduce the number of features by a magnitude or more to eliminate noise from additional features (SNVs and genes with no or little impact, in our case). We have demonstrated that boosted regression trees can separate SCZ and ASD patients based solely on their WES data. This highlights the role of ML in deciphering the genomic architecture of different diseases with shared genetics.

Next, we identified (networks of) genes that are important for each disease, through hierarchical clustering of genes based on the proportion of cases they shared. Although each of the 151 genes may contribute to SCZ or ASD to some extent, our focus was to find the central group of genes that plays an important role in the majority of our cases. Our clustering method highlighted these genes for SCZ (Fig. 1, Supplemental Table 3) and ASD (Fig. 2, Supplemental Table 4). The dendrograms created based on this approach denote genes mutated concurrently in affected individuals and thus provide information about the networks of genes that appear to be important for each of the diseases targeted. This approach can potentially help address the clinical heterogeneity of each disease. For example, after identifying the central genes for SCZ, these genes can be used to cluster SCZ patients and look for subgroups that could then be characterized based on genetics, clinical features, medication response, or disease progression.

Our bioinformatic analysis and literature review of the identified genes revealed multiple pathways and networks important to SCZ and/or ASD. Focusing on the top 10 genes identified by the two boosted regression trees approaches (Supplemental Table 1), we found that some of them already have evidence in the literature linking them to SCZ and/or ASD.

For example, *KIF13A* is a member of the kinesin superfamily proteins (KIFs), which are important for cellular transport and signal transduction [36]. *KIF13A* is located in a SCZ susceptibility region of chromosome 6p23. A recent study on mice lacking *KIF13A* reported elevated anxiety-related traits through a reduction in the serotonin 5HT(1A)R receptor transport and reduced expression of the receptor in neuroblastoma cells and hippocampal neurons [37]. Another study investigating the mechanism of endosomal recycling revealed that *KIF13A* interacts with the protein complex BLOC-1 and Annexin A2, and that dysfunction of these interactions may underly the pathophysiology of neurological defects associated with SCZ [38]. Of note, a rare disruption of another member of the KIFs, *KIF17*, could also lead to SCZ [39]. No evidence was found supporting the involvement of this gene in ASD.

Fanconi-associated nuclease 1 (*FAN1*), a DNA repair enzyme, is located in the chromosome 15q13.3 locus. A microdeletion in the locus, affecting *FAN1* and six other genes, is associated with increased risk of both ASD and SCZ. Deletion of this region using mice models resulted in increased seizure susceptibility and ASD symptoms among other defects [40]. A study systematically searching for SCZ risk variants identified variants in FAN1, which were associated with both SCZ and ASD [41].

Literature review of the genes revealed evidence for both SCZ and ASD, which is consistent with the gene networks hypothesis of common underlying genetic drivers. At the same time, it is interesting to note that some of the genes we identified do not have a (clear) previous link to SCZ or ASD, suggesting that the approaches described in this manuscript can potentially yield new insights for the genetics of the conditions targeted.

Additionally, we conducted a bioinformatics analysis and literature review of the SCZ and ASD “hub” genes. Pathway enrichment analysis was performed using the ShinyGO tool v0.61 [42] based on the ASD “hub” genes (Supplemental Table 3) and SCZ “hub” genes (Supplemental Table 4) identified. Based on the pathway network plot generated with Gene Ontology (GO) biological processes meeting a false-discovery rate (FDR) less than 0.2 (Supplemental Figures 1 and 2), we identified several themes. For ASD, we identified the following themes: 1) *calcium ion transmembrane transport*, 2) *immune system and inflammation*, 3) *cell projection, neuron maturation and synapse organization*, 4) *retinoid metabolic process*, 5) *actin-related processes*, and 6) *blood and platelet coagulation processes*.

There is evidence that changes in *calcium signaling* may be associated with ASD [43–45]. Similarly, multiple studies support a link of *immune dysfunction and inflammation* to ASD [46–48], while strong evidence exists for a link with *synaptic structures* [49–52]. Upregulation of immune genes and downregulation of synaptic genes was observed in the postmortem brains of idiopathic ASD patients [53, 54]. Recent analyses in larger ASD cohorts of postmortem brain collections showed upregulation of immune-microglia and mitochondrial modules, and downregulation of neuronal and synaptic modules [55].

Furthermore, *actin and microtubule processes* are linked to ASD [56]. Also, alterations in actin dynamics by *actin-binding proteins* and *calcium signaling* messengers is associated with ASD [57]. In contrast to SCZ, ASD is associated with an increase in dendritic spine density in several areas of the brain [50], which is thought to be mainly regulated via postsynaptic actin filaments [57].

Some evidence also exists for a link of ASD to *retinoid and retinoic acid metabolic processes* [58, 59], as well as abnormalities in *platelet and coagulation pathways* [60–62].

As illustrated above, several of the themes identified have evidence for a joint role in ASD. In support to this, in Fragile X, a well-known syndrome associated with ASD, evidence has been published for all pathways mentioned above: from dysregulation of calcium signaling, synaptic structures, actin to inflammation, and changes in the retinoid and coagulation pathways [63–69].

For SCZ, our pathway enrichment analysis identified the following themes: 1) *ion transmembrane transport/neurotransmitter transport*, 2) *microtubule/cytoskeleton*, 3) *response to carbohydrates/glucose/hexose stimulus*, and 4) *kidney/renal system development*. There is robust evidence in the literature for the role of *neurotransmitters* in SCZ [70, 71]. Moreover, recurrent evidence exists linking *microtubules/cytoskeleton* and SCZ [72–78]. There is not much evidence for *kidney development* and SCZ, but there have been studies showing that SCZ is associated with chronic kidney disease, even after controlling for demographic, behavioral, and medical risk factors [79, 80]. Furthermore, a study found a polygenic signature differentiating SCZ from controls, which could also significantly differentiate type 2 diabetes patients from controls by predicting a glycemic control indicator, supporting a molecular commonality between SCZ and type 2 diabetes [81]. Of note, *Glucose metabolism* has been shown to be impaired in patients with first-episode SCZ [82] and in antipsychotic-naïve patients with psychosis [83].

## Conclusion

We first showed that supervised learning can distinguish SCZ and ASD patients with high accuracy based solely on their rare SNVs in 151 genes. Through clustering analysis of these genes, we highlighted the important “hub” genes contributing to SCZ or ASD. Bioinformatic analysis revealed several biological themes associated with the “hub” genes of each disorder, including calcium ion transmembrane transport, immune system/inflammation, synapse organization, and retinoid metabolic process for ASD versus ion transmembrane transport, neurotransmitter transport, and microtubule/cytoskeleton processes for SCZ. Our findings demonstrate the usefulness of ML analysis of exome data in the study of the genetic architecture of distinct, yet genetically overlapping, diseases with complex inheritance.

## Future directions

In addition to *rare* SNV, *common* variants [84] and copy number variations (CNVs) also have support in the literature for a role in ASD [85, 86]. Similarly, de novo CNVs [13, 14] and common variants have also been associated with SCZ [87, 88]. The presence of CNVs contributing to these conditions suggests that it would be beneficial for future studies to focus on whole-genome sequencing (WGS) data, thus capturing both SNVs and CNVs, for ML analyses. Moreover, the presence of common variants as contributing factors to SCZ and ASD suggests that we should not only focus on rare variants but also factor in common variants in future ML analyses.

## Supplementary information

**Additional file 1: Supplemental Table 1.** Merged top 10 genes from the *SNV-based* model and top 10 genes from the *gene-based* model. **Supplemental Table 2.** Overlapping genes from the *SNV-based* model and the *gene-based* model. **Supplemental Table 3.** Cluster genes from gene clustering with SCZ patients. **Supplemental Table 4.** Cluster 2 genes from gene clustering with ASD patients.

**Additional file 2: Supplemental Figure 1.** Network plot of significant Gene Ontology biological processes for ASD. **Supplemental Figure 2.** Network plot of significant Gene Ontology biological processes for SCZ.

## Data Availability

The SCZ data used in the preparation of this manuscript were obtained from the Database of Genotypes and Phenotypes (dbGaP) after McGill IRB approval. Raw data used is available in study phs000473.v1.p1. The ASD dataset used in the preparation of this manuscript was obtained from the NIH supported National Database for Autism Research (NDAR). Raw data (including VCF files and additional data files) accessed and used in the preparation of this study is available from NDAR (doi: 10.15154/1169318; doi: 10.15154/1169195).

## Abbreviations

ASD: Autism spectrum disorder
CNV: Copy number variation
DNM: De novo mutation
GBM: Gradient boosted machine
MAF: Minor allele frequency
ML: Machine learning
SCZ: Schizophrenia
SNP: Single nucleotide polymorphism
SNV: Single nucleotide variant
WES: Whole Exome Sequencing
WGS: Whole Genome Sequencing
