# Supplementary material for: Machine learning analysis of exome trios to contrast the genomic architecture of autism and schizophrenia
Source: BMC Psychiatry. 2020 Feb 28;20:92. doi: 10.1186/s12888-020-02503-5 (PMC7049199; doi:10.1186/s12888-020-02503-5)
Supplement: Supplementary file 1 — Additional file 1: Supplemental Table 1. Merged top 10 genes from the SNV-based model and top 10 genes from the gene-based model. Supplemental Table 2. Overlapping genes from the SNV-based model and the gene-based model. Supplemental Table 3. Cluster genes from gene clustering with SCZ patients. Supplemental Table 4. Cluster 2 genes from gene clustering with ASD patients. [file 12888_2020_2503_MOESM1_ESM.doc]

**Supplemental Table 1. Merged top 10 genes from the *SNV-based* model and top 10 genes from the *gene-based*** model.

| **Genes (n=16)** |
| --- |
| SARM1; QRICH2; AKAP1; PCLO; TSPO2; ABCC3; KIF13A; FAN1; CCDC155; PRPF31; SEC24D; SCN4A; CACNA1S; CDSN; HERC2; MUC16 |

Supplemental Table 1. The top 10 genes from the variable importance ranking of the *SNV-based* model and the top 10 genes from the variable importance ranking of the *gene-based* model were combined, and any duplicated genes are removed to create the merged list of 16 genes for subsequent literature review.

**Supplemental Table 2. Overlapping genes from the *SNV-based* model and the *gene-based*** model.

| **Genes (n=151)** |
| --- |
| AGRN; KLHL5; CLCN1; SPRED2; PTPN14; CLECL1; ADAMTS10; PDHX; ENGASE; PRPF38B; DCHS2; UTRN; UBR1; TRAP1; CA1; CYSLTR2; CFTR; LMNTD2; XIRP2; CUL9; NFXL1; ASMTL; DPP7; TACC2; LRRC14B; EFHC1; RAPGEF6; GPR179; KLK14; ABCA7; CHD6; ADPRHL1; MERTK; UGGT2; CTAGE5; SCN4A; ANO9; CACNA1S; DNAH11; CMYA5; LAMC3; CX3CR1; HEBP2; CARS; PIWIL4; SLC29A2; CCNJL; CEP350; ZBTB20; ABCC2; SLC7A14; CD93; ARMC12; ABCA2; PRPF31; NAA38; MTHFD1; KIF7; SEC24D; ABCA13; PLEKHA7; ADCK2; BIRC6; ABCC3; ASPH; MAGEA11; TSHZ3; ZAN; QRICH2; ADGRF1; ATM; DNAH7; PLXNA2; GRIN3B; PHLDB2; ANKRD60; PDZD2; LAMA5; PLCG2; OR6Q1; ZNF429; ANK3; SON; FAM166A; SLC41A3; DCHS1; CCDC8; DNAH10; NTSR1; C8orf58; EPX; C8orf76; GPATCH8; TSPO2; ABCA4; ADAM28; KANSL3; BOD1L1; NOSTRIN; AUNIP; CDSN; MYBPC2; PCLO; SSPO; AGT; KIFC1; ADCY7; CHD1L; CCDC155; KCNG4; NAV3; NLRP11; AMPH; CSF3R; CD109; ABCB4; CAGE1; KIF13A; BAIAP3; ELP4; EML3; DSP; TLR10; KRT71; MRPS15; NID2; LRIG1; ABCG8; AHNAK2; FRAS1; SEC14L4; HCLS1; MED1; ABCA5; ZNF721; AIM1; KMT2C; SEMA4B; CX3CL1; FAN1; AKAP1; HERC2; SAMD11; MEFV; DDX49; AADACL2; SARM1; APLP1; C1orf127; ADAMTS2; TTC31 |

Supplemental Table 2. All SNVs used by the *SNV-based* were mapped to corresponding genes. All genes used by the *gene-based* model was extracted. The gene lists from both models were intersected to get a set of 151 genes used by both models to use for subsequent pathway analyses and literature review.

**Supplemental Table 3. Cluster genes from gene clustering with SCZ patients.**

| **Genes (n=67)** |
| --- |
| AGRN; KLHL5; CLECL1; PDHX; PRPF38B; DCHS2; UBR1; TRAP1; CFTR; LMNTD2; XIRP2; CUL9; ASMTL; TACC2; GPR179; MERTK; CTAGE5; CACNA1S; CMYA5; CX3CR1; CARS; PIWIL4; CEP350; ZBTB20; ABCC2; ABCA2; PRPF31; ABCA13; BIRC6; ASPH; QRICH2; DNAH7; PDZD2; LAMA5; PLCG2; OR6Q1; ANK3; SON; FAM166A; SLC41A3; DCHS1; CCDC8; DNAH10; NTSR1; C8orf58; EPX; ABCA4; BOD1L1; MYBPC2; SSPO; KIFC1; CHD1L; CCDC155; NAV3; NLRP11; CD109; BAIAP3; NID2; AHNAK2; FRAS1; SEC14L4; HCLS1; ZNF721; KMT2C; HERC2; APLP1; TTC31 |

Supplemental Table 3. Genes identified by both the SNV-based and gene-based supervised algorithms were clustered. The proportion of overlapping mutated SCZ probands between each pair of genes is used as the similarity metric for clustering. We obtained 3 clusters of genes*.* The cluster of genes with the highest ratio of number of SCZ cases represented per gene (506 out of a total of 598 patients represented by 67 genes) is shown in this table.

SCZ: Schizophrenia

**Supplemental Table 4.** Cluster 2 genes from gene clustering with ASD patients.

| **Genes (n=38)** |
| --- |
| AGRN; UTRN; TRAP1; TACC2; MERTK; SCN4A; CMYA5; CARS; SEC24D; ABCA13; PLEKHA7; ZAN; QRICH2; DNAH7; PDZD2; LAMA5; PLCG2; ZNF429; EPX; ABCA4; BOD1L1; MYBPC2; PCLO; SSPO; KIFC1; NAV3; DSP; NID2; AHNAK2; FRAS1; AIM1; KMT2C; CX3CL1; FAN1; AKAP1; HERC2; MEFV; SARM1 |

Supplemental Table 4. Genes identified by both the SNV-based and gene-based supervised algorithms were clustered. The proportion of overlapping mutated ASD probands between each pair of genes is used as the similarity metric for clustering. We obtained 2 clusters of genes*.* The cluster of genes with the higher ratio of number of ASD cases represented per genes of (589 out of a total of 598 patients represented by 38 genes) is shown in this table.

ASD: Autism spectrum disorder
