# Supplementary material for: Machine learning analysis of exome trios to contrast the genomic architecture of autism and schizophrenia
Source: BMC Psychiatry. 2020 Feb 28;20:92. doi: 10.1186/s12888-020-02503-5 (PMC7049199; doi:10.1186/s12888-020-02503-5)
Supplement: Supplementary file 2 — Additional file 2: Supplemental Figure 1. Network plot of significant Gene Ontology biological processes for ASD. Supplemental Figure 2. Network plot of significant Gene Ontology biological processes for SCZ. [file 12888_2020_2503_MOESM2_ESM.doc]

**Supplemental Figures**

**
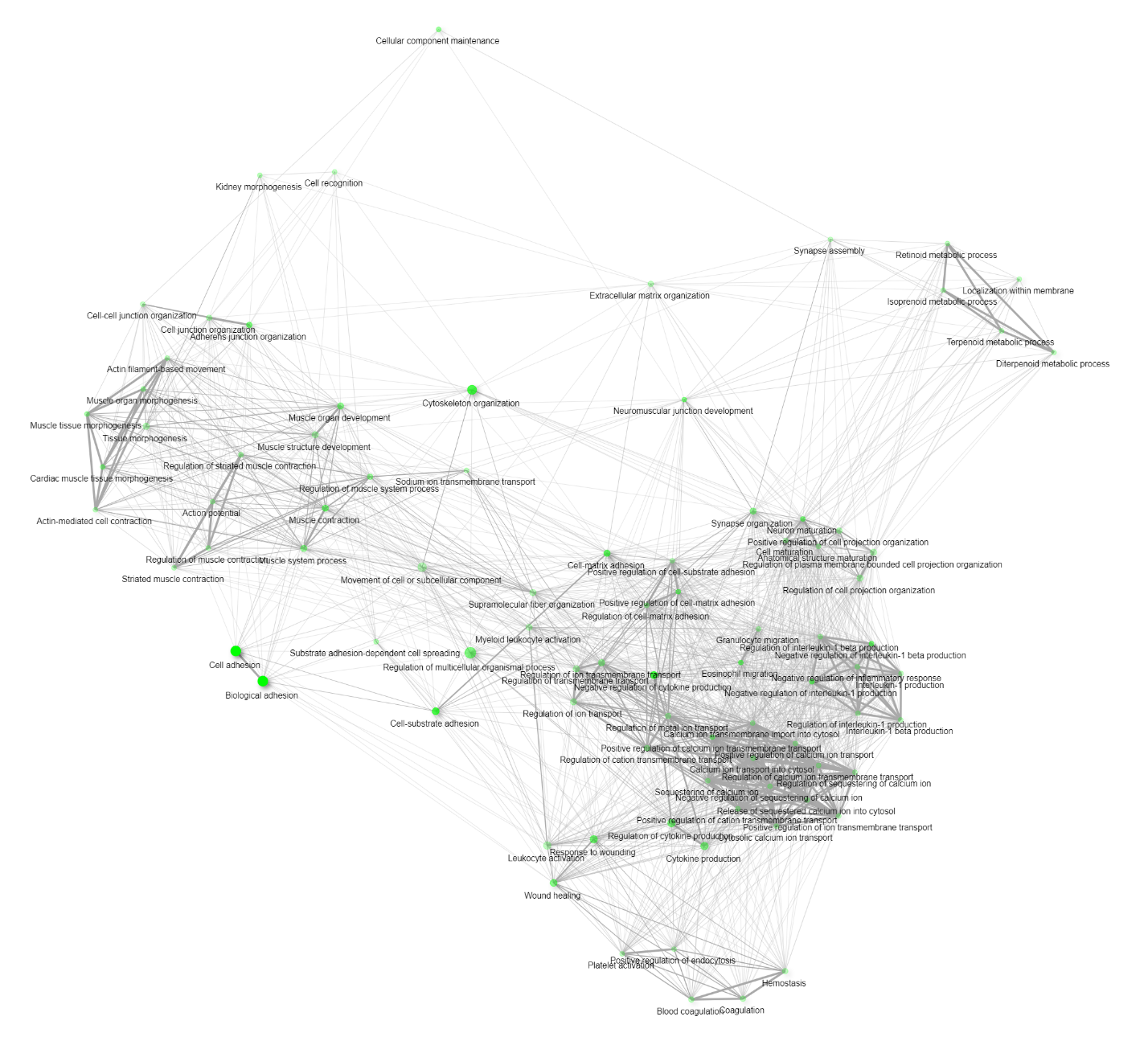
**

**Supplemental Figure 1. Network plot of significant Gene Ontology biological processes for ASD.**

Using ASD “hub” genes from Supplemental Table 3, a pathway enrichment analysis was performed using the ShinyGO tool v0.61. A network plot was generated for Gene Ontology (GO) biological processes meeting a false-discovery rate of less than 0.2. Pathways (nodes) are connected if they share 20% or more genes. Thicker edges represent more overlapped genes. Important pathway themes were identified based on regions of densely interconnected nodes.

ASD: Autism spectrum disorder


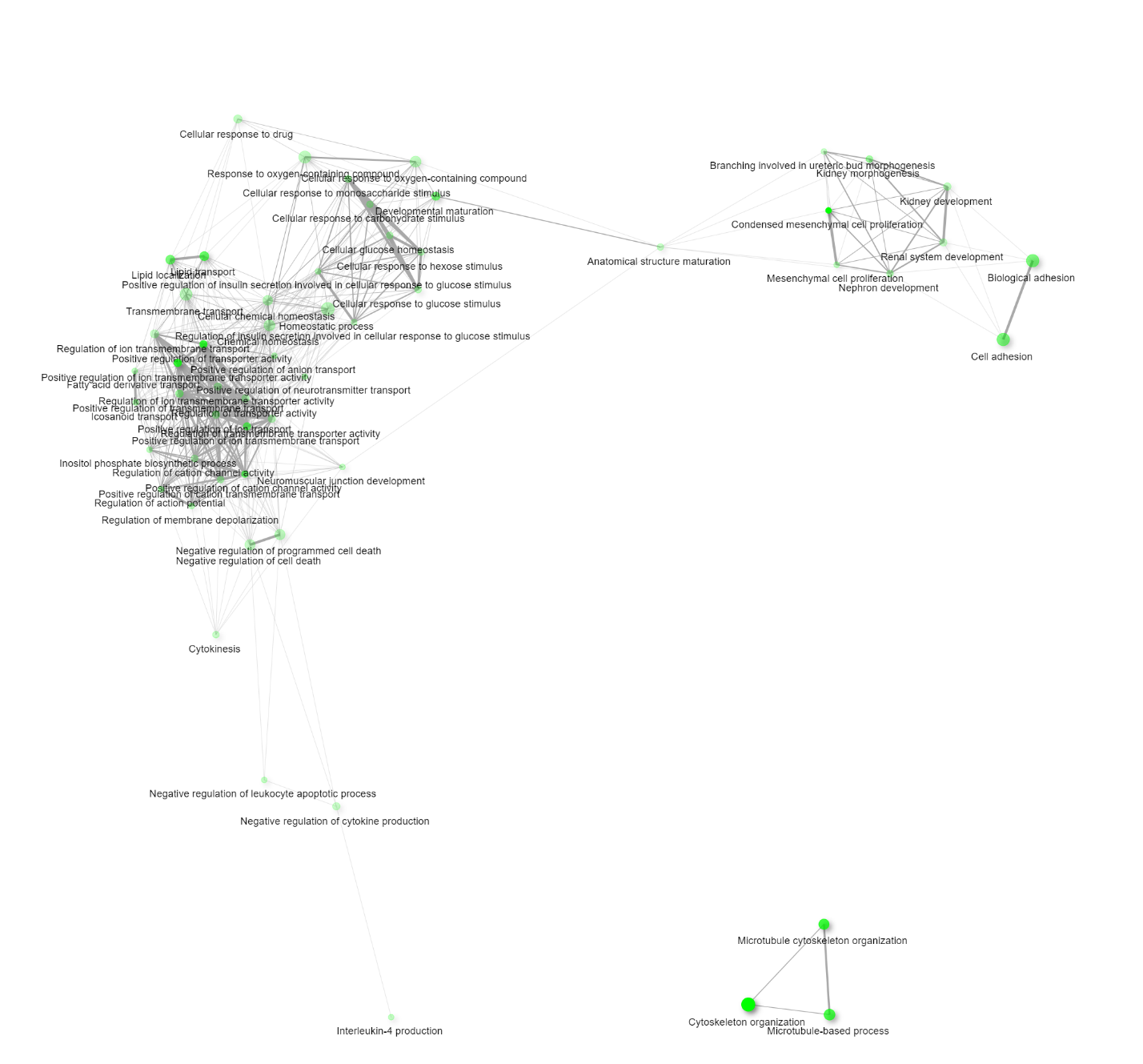


**Supplemental Figure 2. Network plot of significant Gene Ontology biological processes for SCZ.**

Using SCZ “hub” genes from Supplemental Table 4, a pathway enrichment analysis was performed using the ShinyGO tool v0.61. A network plot was generated for Gene Ontology (GO) biological processes meeting a false-discovery rate of less than 0.2. Pathways (nodes) are connected if they share 20% or more genes. Thicker edges represent more overlapped genes. Important pathway themes were identified based on regions of densely interconnected nodes.

SCZ: Schizophrenia
